# Going beyond the fifth child: Exploring the determinants of desire for more children among high parity partnered women in Uganda

**DOI:** 10.1371/journal.pgph.0004730

**Published:** 2025-06-25

**Authors:** Godfrey Tumwizere, Yiga Joseph Douglas, Allen Kabagenyi, Betty Kwagala

**Affiliations:** 1 Department of Community Health and Behavioral Sciences, School of Public Health, Makerere University, Kampala, Uganda; 2 Department of Population Studies, School of Statistics and Planning, Makerere University, Kampala, Uganda; PLOS: Public Library of Science, UNITED STATES OF AMERICA

## Abstract

**Background:**

Despite the extensive research on fertility desire among women worldwide, there is a dearth of literature on the desire for more children among high-parity women. This study aimed to identify the determinants of the desire for more children among high parity partnered women in Uganda.

**Methods:**

This study was based on nationally representative data from the 2016 Uganda Demographic and Health Survey. The study sample comprised of a weighted sample of 4502 women aged 15–49 years with five and more children. A complimentary log-log model was fit to identify factors associated with the desire for more children among high-parity women in Uganda at the 5% level of significance.

**Results:**

The findings revealed that 21% of high parity partnered women desired more children. The odds of desire for more children were 26% higher among women without decision-making autonomy on the number of children (AOR = 1.262 95% CI 1.109-1.415) than women with decision-making autonomy, women with preference of more than 3 boys had 2 times odds of desire for more children compared to those who preferred less than three boys (AOR = 2.021, 95% CI = 1.726-2.367) while Catholic women had 80% higher odds of desire for more children (AOR = 1.896, 95% CI = 1.786-2.020) compared to Anglicans. On the other hand, the odds of having a desire for more children were 33% lower among Muslims (AOR = 0.676, 95% CI 0.559–0.817) and 21% lower among Pentecostals (AOR = 0.70895% CI = 0.598–0.837) compared to Anglicans. The odds of having a desire for more children were 75% lower among women with primary education (AOR = 0.252, 95% CI 0.062-0.441) than among those with no education. Compared to women using modern contraceptives the odds of desire for more children were 38% lower (AOR = 0.620, 95% CI 0.481- 0.865) than those with not using modern contraceptives. Women with at least five living children had 75% lower odds of desire for more children (AOR = 0.255, 95% CI 0.060-0.549) compared to those with two living children. Women with primary education had 75% reduced odds of desire for more children compared to no education (AOR = 0.252 95% CI 0.062-0.441) while women whose husbands attained at least a secondary level of education had 79% reduced odds of desire for more children (AOR = 0.210, 95% CI 0.190-0.411) compared to women whose husbands had no education.

**Conclusions:**

This study recommends that Uganda’s policymakers and Programme implementers emphasize the attainment of secondary or higher education, collaborate with religious leaders to promote health education sensitive to religious beliefs and practices and empower women to challenge social norms that restrict women’s decision-making autonomy on fertility.

## Introduction

The global population of over eight billion people is projected to reach nearly 9.6 billion by 2050 [1]. Africa’s population is expected to grow from 1.5 billion in 2024 to 2.4 billion in 2050, accounting for over 60% of the projected global population growth during this period [1,2]. While fertility rates have declined in many regions, some low- and middle-income countries still experience high levels of fertility [3]. Among the 78 million people added to the world population annually, approximately 97% originate from low- and middle-income countries [4]. Scientists predict that the continuous population growth will reach a level where there will not be enough resources to sustain life, and this raises concerns about whether the earth is facing overpopulation [5].

Sub-Saharan Africa with the lowest world economies have youngest population with 40% of the population younger than 15 years [6]. Studies reveal that in sub-Saharan Africa, women still produce more than four children [4,7], but more importantly, many women with as many as five children still want to produce more [4]. Uganda has the second youngest population in the world, with more than three-quarters (78%) aged below 35 years. Demographers project this youthful population to double in the next 25 years [8]. Despite the decline in Uganda’s fertility from 6.9 children to 5.2 children per woman [9] the current fertility level raises concerns about the potential impact on the country’s ability to achieve the desired fertility [10,11]. A significant proportion of high-parity women with five or more children who apparently should have attained the desired fertility, continue to express a desire for more children or are undecided whether to have more [4]. This not only defies conventional expectations but also perpetuates high fertility rates, exacerbates dependency ratio that strain already limited resources potentially hindering the realization of demographic dividend which is a critical component of sustainable development [12,13].

Research on fertility desires among high-parity women show several factors influencing their fertility preferences. A study by Atake and Gnakou Ali [7] in four high-fertility countries in sub-Saharan African countries found that women’s empowerment in economic and familial domains, is associated with a desire for fewer children. Elements such as education, decision-making power, and control over household resources are significant in shaping fertility preferences [7]. Similarly, Ahinkorah, Seidu [4] analyzed data from 32 Sub-Saharan African countries and identified that age, educational attainment, contraceptive use, and the number of living children significantly influence women’s desire for more children. Women with higher education levels and those using contraceptives were less likely to desire additional children [4]. In Ghana, research by Owoo [14] highlighted that internalized patriarchal norms and perceptions of male partner pronatalism are linked to women’s intentions to have more children, which emphasizes the role of socio-cultural factors in fertility decisions [14]. Furthermore, a synthesis of longitudinal studies across 28 countries in Africa and Asia demonstrated that while women’s desire to stop childbearing is a strong predictor of subsequent fertility, discrepancies often exist between fertility preferences and actual behavior [15].

In Uganda, a number of studies [16–21] have explored the factors affecting the desire for more children in different settings. Some studies have been conducted among HIV-positive individuals and HIV-discordant couples [20,22], whereas other studies have explored fertility desire from a general population perspective, including among married or cohabiting individuals [11]. These studies indicate that the desire for few children is still low [17,18,20]. The limited information on desire and intention to have more children among women with five or more children, leaves a noticeable gap in the literature that calls for an in-depth examination. This study aimed to determine factors associated with the desire for more children among high parity partnered women. The study attempted to answer the following research question; What are the determinants of the desire for more children among women with 5 + children? We hypothesized that age, level of education and decision-making autonomy on the number of children do not significantly affect the desire for more children among high parity partnered women in Uganda.

## Methods

### Data source

The study used secondary data from Uganda Demographic and Health Survey (UDHS). Data were accessed with permission from Measure DHS. UDHS is a cross-sectional survey that uses a stratified two-stage cluster sampling design. The 2016 UDHS interviewed 18506 women aged 15–49. We selected 4502 partnered women who reported five and more children during the survey.

### Measure of the outcome variable

The outcome variable “desire for more children” was derived from the question, “Would you like to have a (another) child, or would you prefer not to have any more children?”. The question had five response options: “want another child,” “want no more,” “cannot become pregnant,” “undecided,” and “don’t know”. Women who responded that they wanted another child were considered to have a desire for more children, whereas others were considered not to have a desire for more children. Thus, the variable was recorded on a binary scale of 0 = does not desire more children and 1 = desires to have more children. Women who provided any other response (“cannot get pregnant,” “undecided,” or “don’t know”) were excluded because their responses were unclear about their fertility preference.

### Measure of explanatory variables

The explanatory variables for this study included age, education, place of residence, wealth status, employment status, religion, knowledge of family planning, number of living children, mortality experiences, age at first sex, preference of boy child, use of contraceptives, history of pregnancy termination, ideal number of children, place of delivery, age at first cohabitation, partner employment status, partner education, husband desire for children and decision-making autonomy. There were only 8 women aged 15–24 with at least 5 children and were regrouped by 20–29 years. The selection of these explanatory variables was based on previous studies and their hypothetical relationship with the desire to have more children.

### Statistical analysis

Statistical analysis was performed using STATA (Version 17) [23]. Data were weighted to ensure representativeness of the study population. The analyses were performed at three levels. At univariate analysis, the frequency and percent distribution of women according to the sociodemographic characteristics were determined. At the bivariate level, a cross tabulation of desire for more children and each explanatory variable was performed. The Pearson chi square test was used to measure the significance of associations of desire for more children with each explanatory variable at 5% level of significance. All variables with a ρ value less than or equal to 0.05 and with no autocorrelation (Vif < 10) were automatically considered for further analysis at the multivariate level. Age, wealth index and husband’s desire for more children showed autocorrelation with Variance inflation factor greater than 10 and were not considered for further analysis. At the multivariate level, since desire for more children was measured on a binary scale (1 = desires and 0 = does not desire), a complementary log-log regression model was used. Model building was done using stepwise selection to fit the factors associated with the desire for more children and results presented in the form of adjusted odds ratios (AORs) with 95% confidence intervals.

## Results

### Characteristics of high parity partnered women in Uganda

Table 1 presents the distribution of women’s sociodemographic characteristics. The results indicate that 21% of high-parity women desire to have more children. More than half (51%) of the high-parity women who desired more children were aged 30–39 years, the majority (87%) resided in rural areas, more than half (66%) had attained a primary level of education, 41% were affiliated with the Catholic religion, 85% were currently working, more than half (67%) had never experienced pregnancy complications, and 54% preferred at least 3 boys. In addition, more than half (66%) of the women were aware of a family planning method, and 93% had a history of child mortality. With respect to wealth, the findings show that almost half (48%) of the high-parity women were from poor wealth category households, 45% were from the middle quintile, and 7% were from the rich category. The results also indicate that the majority (85%) of the high-parity women were working and 87% had at least 5 living children. The results also revealed that 59% had never used contraceptives, more than half (57%) had their first cohabitation between 15 and 19 years, whereas approximately one quarter (19%) had their first cohabitation at before 15 years. Concerning age at first sex, more than half (82%) had their first sex between 15 and 19 years, and 12% had their first sex below 15 years. Seventy per cent of the women had no history of pregnancy termination. In terms of partner characteristics, 95% of women reported that their partners were working, 52% of women had partners with primary education, approximately 3 in 4 women (74%) had partners who desired more children, and 65% of women did not decide on matters regarding the number of children.

**Table 1:** Distribution of high parity partnered women by selected characteristics, UDHS 2016.

Characteristic	Frequency (n = 4502)	Percent (%)
Desire for more children		
Desires to have more children	1053	21.4
Doesn’t desire to have more children	3449	76.6
Age		
20-29	476	10.6
30-39	2323	51.4
40-49	1713	38.0
Place of residence		
Urban	599	13.3
Rural	3903	86.7
Education level		
No education	1044	23.2
Primary	2953	65.6
Secondary+	505	11.2
Religion		
Anglican	1442	32.0
Catholic	1843	40.9
Muslim	493	11.0
Pentecostal	74	1.6
Other	650	14.4
Wealth		
Poor	2172	48.3
middle	2016	44.8
Rich	314	6.9
Covered by Health Insurance		
No	4455	98.9
Yes	47	1.1
Current working status		
Not working	672	14.9
Working	3830	85.1
Number of living children		
0–2 Children	20	0.4
3–4 Children	574	12.8
5 + children	3908	86.8
Knowledge of any family planning method		
No knowledge	1544	34.3
Has knowledge	2958	65.7
Pregnancy complications		
Never had	3031	67.3
Ever had	1471	32.7
Age at first sex		
below 15	530	11.6
15-19	3625	81.6
20-24	277	6.2
25 above	18	0.4
Age at first cohabitation		
below 15	847	18.8
15-19	2560	56.9
20-24	784	17.4
25 above	311	6.9
Use of contraceptives		
Not using	2665	59.2
Using contraceptives	1837	40.8
Preferred number of boy children		
< 3 Boys	2066	45.9
3–4 Boys	1801	40.0
5 + boys	635	14.1
Husbands Employment status		
Not working	232	5.2
Working	4270	94.8
Husbands Education		
No education	475	10.6
Primary education	2540	52.4
Secondary +	1487	33.0
Husbands desire for more children		
Yes	3314	73.6
No	1188	26.4
Decision-making autonomy concerning the number of children		
Decides alone	1556	34.6
Doesn’t not decide alone	2946	65.4

### Bivariate analysis of the factors associated with the desire for more children

The findings in Table 2 reveal that the desire to have another child decreased with increasing age, and the association between the woman’s age and the desire for more children was significant (P = 0.000). More than half (approximately 57%) of the women who desired more children were aged 20–29 years, with 26% aged 30–39 years and 6% aged 40–49 years. The woman’s working status (ρ = 0.030) was significantly associated with the desire for more children. The desire for more children increased with increasing education levels. More than 1 in 5 women (23%) with secondary education and above had a desire for more children, with 19% of women without education having a desire for more children. The preferences of the boy child (ρ = 0.000), religion (ρ = 0.024), number of living children (ρ = 0.000), partner desire (ρ = 0.000) and decision-making autonomy (ρ = 0.000) were statistically significantly associated with the desire for more children.

**Table 2:** Desire for more children by selected characteristics.

Variable	Desire for more children (n = 4502)		P value	Chi (χ^2^), DF = 659
	No (%)	Yes (%)		
Age				
20-29	42.9	57.1	**0.000** ^*^	5.445
30-39	71.6	28.4		
40-49	94.2	5.8		
Place of residence				
Urban	80.5	19.5	0.100	4.30
Rural	76.8	23.2		
Education level				
No education	80.5	19.5	**0.026** ^*^	11.061
Primary	76.5	23.5		
Secondary+	76.8	23.2		
Religion				
Anglican	78.5	21.5	**0.024** ^*^	20.332
Catholic	78.5	21.5		
Muslim	70.5	29.5		
Pentecostal	76.2	23.8		
Other	77.8	22.2		
Wealth				
Poor	74.8	25.2	**0.007** ^*^	8.049
Middle	77.9	22.1		
Rich	80.4	19.6		
Current working status				
Not working	76.9	23.1	**0.030** ^*^	11.062
Working	77.4	22.6		
Boy child preference				
0-2	82.5	17.5	**0.000** ^*^	14.865
3-4	71.8	28.2		
5+	76.1	23.9		
Number of living children				
0-2	46.9	53.2	**0.000** ^*^	17.493
3-4	62.7	37.3		
5+	82.3	17.8		
Last child alive				
No	78.4	21.6	0.698	0.183
yes	79.4	20.6		
Covered by health insurance				
No	79.4	20.6	0.787	0.101
yes	77.7	22.3		
Pregnancy termination				
Never terminated	76.6	23.4	0.085	2.783
Ever terminated	79.2	20.8		
Use of contraceptives				
Not using	75.9	24.1	**0.020** ^*^	0.029
using	79.4	20.6		
Place of delivery				
Home	73.2	26.8	0.238	4.718
Health facility	71.2	28.9		
Others	62.2	37.8		
Age at first cohabitation				
Below 15 years	79.9	20.1	0.092	5.172
15–24 years	75.8	24.2		
Above 25 years	78.0	22.0		
Partners Education				
No education	73.6	26.4	0.163	5.005
Primary education	77.9	22.1		
Secondary+	77.9	22.1		
Husband’s desire for more children				
Wants	81.0	19	**0.000** ^*^	8.098
Doesn’t want	75.4	24.6		
Decision-making autonomy on the number of children				
Decides	81.1	19.0	**0.000** ^*^	18.497
Does not decide	75.4	24.6		

* *p < 0.05*.

### Factors associated with the desire for more children among high parity partnered women

Table 3 shows the results of the multivariate complementary log-log of desire for more children. The desire for more children among high-parity women was associated with education level, religion, number of living children, preference for boy children, partner education, decision-making autonomy and use of contraceptives.

**Table 3:** Complementary log–log regression of factors associated with the desire for more children.

Variable	AOR	P value	95% CI
Education level			
No education^*^	1.000		
Primary	0.252	**0.009**	0.062-0.441
Secondary+	0.208	*0.159*	0.081-0.497
Religion			
Anglican^*^	1.000		
Catholic	1.896	**0.037**	1.786-2.020
Muslim	0.676	***0.000***	0.559-0.817
Pentecostal	0.708	***0.000***	0.598-0.837
Other	0.701	0.158	0.427-1.149
Number of living children			
0–2 Children^*^	1.000	.	
3–4 Children	0.288	*0.434*	1.135-1.590
5 + children	0.255	**0.001**	0.060-0.549
Boy child preference			
0–2 boys^*^	1.000		
3–4 boys	2.021	***0.000***	1.726- 2.367
5 + boys	1.964	***0.000***	1.580- 2.440
Husbands’ education			
No education^*^	1.000		
Primary education	0.264	0.118	0.067-0.596
Secondary +	0.210	***0.022***	0.190-0.311
Decision-making autonomy on the number of children			
Decides^*^	1.000		
Doesn’t decide	1.318	***0.001***	1.129-1.540
Use of modern contraceptives			
Not using^*^	1.000		
Using	0.620	***0.021***	0.481-0.865

*Bold italics indicate significant values at α = 0.05 (p < 0.05, CI is 95%*

* *indicates the reference category).*

The likelihood of the desire for more children decreased with increasing education level. Women with primary education had 75% lower odds of desire for more children (AOR = 0.252, 95% CI = 0.062-0.441, whereas women with at least secondary education had 79% lower odds of desire for more children (AOR = 0.208, 95% CI = 0.081-0.497) than women with no education. Similarly, women whose partners had at least secondary education had 79% reduced odds of desire for more children (AOR = 0.210, 95% CI = 0.190-0.311) compared than women whose partners have no education. Compared with Anglican women, Catholic women had 89% increased odds of desire for more children (AOR = 1.896, 95% CI = 1.786 - 2.020), whereas Muslim women had 33% reduced odds (AOR = 0.676, 95% CI = 0.559-0.817) of desire for more children. In the same vein, the women who said that they were Pentecostals had 29% reduced odds (AOR = 0.708, 95% CI = 0.598-0.837) relative to their Anglican counterparts. Women with at least five living children had 75% reduced odds of desire for more children compared to those with less than three living children (AOR = 0.255, 95% CI = 0.060-0.549). Additionally, women who used contraceptives were 0.6 times less likely (AOR = 0.620, 95% CI = 0.481-0.865) to desire more children than women who did not use contraceptives. Compared with those with no education, the odds of desire for more children decreased with education level. On the hand On the other hand, decision-making autonomy concerning the number of children and the preference of boy children increased the odds of the desire for more children., Findings indicate that women who preferred 3–4 boys were 2 times more likely (AOR = 2.021, 95% CI = 0.1.726-2.367) and that those who preferred 5 + children were 1.9 times more likely (AOR = 1.964, 95% CI = 1.580-2.440) to desire more children than those who preferred 0–2 boys.

## Discussion

Approximately one in five (21%) high-parity women desired to have more children. The study revealed that having no education was positively associated with the desire for more children. The odds of desire for more children among women with primary education and secondary education were lower than those among women with no education. This finding is similar to those of other studies in Uganda [20,24,25] and Ghana [26], Ethiopia [27] other countries in sub-Saharan Africa [11,28]. This finding suggests that educated women are exposure to health messages, understand the importance of small family size and more likely to make informed choices about the number of children they want to have and prioritize providing better opportunities for their existing children rather than having more. This finding, however, contrasts with Matovu, Makumbi [20] who found that in Uganda, women with primary education had greater desire for additional children compared to those with no education, a pattern attributed to cultural norms and limited socio-economic mobility despite minimal schooling [20]. Similarly, analysis across sub-Saharan Africa by Channon and Harper [29] revealed that education alone may not uniformly reduce fertility desires but its impact is mediated by factors like quality of schooling, exposure to reproductive health services, economic opportunities, and entrenched gender and cultural norms [29].

The findings indicated that catholic women had increased odds of desire for more children compared to Anglicans. This can be linked to the fact that Catholic Church promotes openness to life in marriage and discourages modern contraception. The catholic doctrine emphasizes that children are blessings, and procreation is a central purpose of marriage hence each marital act should remain open to the possibility of life. This finding is similar to other studies in Uganda [20,25]. Muslim and Pentecostal women were less likely to desire more children than Anglican women. Religion has immense significance in most societies and thus plays an important role in promoting acceptance or creating resistance to family planning. This finding is in line with a study performed in Nepal [30,31], which showed that Muslim and Pentecostal women were less likely to desire more children than Anglican women. This finding contradicts the findings of other studies in Ghana [26] Ethiopia [27], Kenya [32] and Tanzania [33], which have shown that Anglican women have less desire for more children than Muslim women.. This is explained by the fact that religious tradition strongly influences the uptake of family planning, with a wide range of interpretations of most religious traditions affecting the perceived acceptability of family planning programs.

The results showed that women with five or more living children were less likely to desire more children. This is because women with five or more living children may be satisfied with their ideal number of children. This finding is consistent the previous study conducted in Uganda comparing predictors across regions and revealed that women with more children are satisfied with their fertility and are more likely to use contraceptives to limit the fertility [34] and another study conducted in 33 countries on marital satisfaction, sex, age, marriage duration, religion and desire for more children which found that women with more than 4 children have less desire for more children [35]. This is partly because, these women may prioritize quality over quantity, focusing on providing the best care and opportunities for their existing children rather than expanding their family further.

Similarly, Women with three or more boys were less likely to desire more children than women with fewer boys. This finding agrees with the findings in Nigeria, Asia and India. In Asia women opt for sex selective abortion in favor of a producing a boy child [36]. Similarly women in India women desire for more child if the first child born is a girl [37]. This is explained by the fact that women with several boys might be contented with family dynamics and not feel the need for additional children. This, however, contrasts with the findings of a study in Taiwan on the value of children, sex preference and fertility desire among young Taiwanese women [38], which showed that the desire for children was associated with the balanced sex composition of children and Iran [39] that showed preference of sex had no significant effect on fertility.

Women who use contraceptives are less likely to desire more children. This is explained by the fact that women who are using contraceptives make informed decisions about family size and might not wish to give birth to additional children. Similar findings consistent with this was reported in Uganda where women who were most women (72%) using modern contraceptives did not intent to have more children in the future [40,41]. The study further revealed that women without decision-making autonomy were more likely to desire more children than their counterparts were. This finding is similar to findings of other studies [42,43] which showed that women with high decision-making autonomy were associated with the desire for no more children. This can be partly explained by the fact that women who lack decision-making autonomy may feel pressured to conform to family expectations or desires and ultimately have more children. Women with limited decision-making autonomy may also face barriers to accessing contraception and family planning services, and in such situations, they may continue to have children because they lack the means to prevent pregnancies.

## Conclusions

The study assessed the critical research gap of desire for more children among high parity women who ideally should have attained the desired fertility. Findings indicate that one in five high-parity women desire to have more children. The desire for more children is significantly associated with education level, religion, number of living children, preference of boy child, decision making autonomy and use of modern contraceptives. The study found evidence to accept the hypothesis that age does not significantly affect the desire for more children. The study also found evidence to conclude that decision making autonomy and education level significantly affect the desire for more children. These findings have implications for future fertility thus recommend that policymakers and Programme implementers should work toward providing education and awareness programs on the benefits of family planning, small family size especially to women with no formal education or primary education. The study also recommends empowering women through increased autonomy and participation in household decisions can lead to more balanced fertility preferences, collaboration with religious leaders to promote health education sensitive to religious values while promoting informed decision making. The study further recommends promotion of gender equality to reduce the desire for more children based on gender preference.

This study examined the determinants of desire for more children using a nationally representative data from Uganda’s major regions. The data used is trustworthy because it was collected by a reputable national organization that conducts large surveys and censuses. However, this study, like other cross-sectional studies, cannot draw causal inferences about the desire for more children. The study only looked at relationships between the selected variables and the desire for more children Therefore, an in-depth analysis of the factors associated with the desire for more children through a qualitative approach and trend analysis between 2001 and 2022 is needed to fully understand the desire for more children among high-parity women in Uganda.
